# Action of Remedies

**Published:** 1878-04

**Authors:** S. W. Johnson

**Affiliations:** Cumming, Ga.


### ACTION OF REMEDIES.

INAUGURAL THESIS FOR GRADUATION IN ATLANTA MEDICAL COLLEGE, MARCH 1, 1878.

By S. W. JOHNSON, Cumming, Ga.

Within the range of the professional pursuits of medical men there is no subject more interesting or more worthy of investigation than that selected for this essay.

For the proper and successful treatment of disease it is absolutely necessary that we have a right understanding, not only of the cause and nature of the pathological conditions to which our remedies are directed, but a correct knowledge of the physiological action of those remdies.

When our acqaintance with these two subjects is complete, we shall be able to do all that man can by any possibility do in allaying human suffering. Without such knowledge of the natural action of remedies, we are unable to combat successfully against our common enemy—disease.

The tempting tendency to study remedies according to the diseases in which they are used, or their therapeutic result—coupling certain remedies with certain diseases—to the exclusion of their physiological action, is quite a difficulty in the progress of rational medicine, and leads to the empirical idea of specifics for the cure of disease.

This system requires for its elaboration a comparatively small degree of knowledge or investigation, hence the exceedingly great number of its votaries.

There is a wide difference between the physiological action of a remedy and the therapeutic result of that action. They are in relation to each other as *cause* and *effect.*

The physiological action, the only true action of a remedy, is that which is exerted upon an organ or tissue, both in a diseased and in a healthy state, and must be understood in order to the rational treatment of disease.

The following instance will serve to illustrate the routine manner in which remedies are often employed: A physician of this city came to the office of my preceptor last summer, to consult him in regard to a case which he said had annoyed him no little. He remarked that it was a case of acute inflammation of the kidneys, bladder, and peri-uterine structure, with consequent painful micturition and scanty, high colored urine. He was asked what course of treatment he had adopted: "I have," said he, "used the oil of turpentine in connection with other diuretics, and my patient, instead of improving, is growing rapidly worse."

The friend whom he was consulting then remarked that he did not wonder the result of such a course of treatment. "The remedies you are using," said he, "will but aggravate and increase the morbid condition which you are attempting to relieve. You do not wish," said he, "to use a stimulent to the already excited and inflamed kidneys, but on the contrary you want a renal sedative; for while oleum terebinthinæ will, under certain conditions, produce diuresis, there are other conditions in which it is not a diuretic 'so-called.'"

The *modus operandi* of remedies can be accounted for in three different ways, viz: 1. On mechanical principles; 2. On chemical principles; 3. On vital principles.

In the human mind there is a tendency to explain everything; and it was but natural in former times, when men knew little of chemistry and physiology, to attempt to account for the action of all remedies, both local and elective, through mechanical process. They claimed that the shapes of the minute particles of remedies are sufficient to account for the several actions.

Locke, in his Essay on the Human Understanding, published in 1689, said: "Did we know the mechanical affections of the particles of rhubard, hemlock, opium, and a man, as a watch-maker does those of a watch, whereby it performs its operations, and of a file, which, by rubbing on them, will alter the figure of any of the wheels, we should be able to tell beforehand that rhubarb will purge, hemlock kill, and opium make a man sleep."

We know that many local remedies act through this process, by protecting denuded and irritable surfaces from the atmosphere and other irritating and foreign substances, or by the unevenness and roughness of its surface tending to irritate the part.

It does not seem to be altogether impossible that the action of some at least of the elective remedies, particularly of those acting upon the nervous system, should depend upon the peculiar mechanical arrangement of the molecules of these substances, as related to those of the tissues which they influence.

We know the nerves are particularly susceptible to mechanical impressions, upon which depend the phenomena of two at least of the five senses—those of hearing and touch. There is no more plausable explanation of this action, and if we accept the atomic theory, by which many chemical phenomena are cleared up and explained, we must admit that the ultimate atoms of all compound bodies, whether fluid or solid, have a peculiar arrangement and shape.

While it is necessary that a remedy which affects the nervous system be absorbed and taken into the circulation in order to make its impress upon the brain and spinal cord, it is admitted that it can impress the nerves themselves, changing or modifying their function without first being taken into the circulation. Can this action be accounted for in any other way than by mechanical principles?

While this is conjecture, to a great extent, it explains many of the phenomena which are otherwise inexplicable.

Like the older, some even of the modern writers have attempted to account for the action of remedies in general through chemical process. Muller thinks that the agency of many remedies may be explained by their chemical affinities. He supposes that they may effect a change in the nutritive fluids, or that they may so disturb the state of combination in which the elements of an organ be, that it becomes insensible to the action of morbid stimuli. Some have tried in this way to explain the action of alcohol by its chemical affinity for the brain.

While some remedies act through chemical process, there are many the action of which cannot be explained in this way. We know that the action of most nerve-medicines, and of gland-medicides cannot be reasonably explained on any such hypothesis.

Chemico-vital force is quite different from chemical force. It must not be imagined that chemical solutions, decomposition, and reactions of every kind are allowed to take effect in the human system as in the laboratory of the chemist; for in the former there are many disturbing and controlling causes which suffice to hold them in check. Yet there are many remedies the action of which cannot be accounted for in any other manner than through chemical process.

Of local remedies acting through this process, we have those affecting adventitious substances and structures, such as anthelmintics, escharotics and antiseptics. Of elective remedies, we have those affecting the blood, such as hæmatinics, spanæmics, diluents, hæmostatics and catalytics.

The reaction produced in the blood and in the fluids of the tissues by these remedies is sufficient to account for their action.

Many of the local remedies and most of the elective remedies affecting solid structures act through vital process. By vital process is meant those changes which are produced in capillary circulation or vitality of the part which are neither mechanical nor chemical, and which can take place only in the living body. As we have said, the most plausible explanation of the action of most remedies is upon this hypothesis. The exact nature or manner of this action, however, seems to us to be but little understood.

There are many theories extant as to the action of these vital or general remedies upon disease. The rule of the disciples of Hahnemann (the homœpathists) is *similia similibus carantur.* Upon this idea, I suppose, they would administer strychnia in tetanus, and opium in congestion of the brain; and perhaps they would decline to treat small-pox or syphilis, because they can find no medicine to produce these affections.

The maxim of Hypocrates was, *Contraria contraris curantur.* Upon this idea, I suppose purgatives alone would be given in constipation, no matter upon what the affection might depend.

Many other theories have been advanced as to the curative effect of remedies, but between these extremes there is a "happy medium," and we know now that there are various ways in which remedies act in the cure of disease.

To sum up the matter, we may say there is but one natural specific action of a remedy, and that is physiological, and that this action may be had through mechanical, chemical or vital process.

Many remedies have two actions, primary and secondary. Primary is the first impression that is made by a remedy upon an organ or tissue. This first impression is often quite transient, giving place to the secondary and usually more important action of the remedy.

The first action produced by opium is stimulation, which lasts but for a short time; this is soon followed by the secondary, which is sedative, and the most permanent action.

Local as well as elective remedies have these two actions. We see this exemplified in the action of the cauterant, the first being stimulant, the second quite the opposite. It is a doubtful question in our mind whether there can be too distinct actions exerted upon the same organ or tissue by the same dose of one remedy. We are of the opinion that what is called the secondary action is but the result of the first or primary action of the remedy. The therapeutic result of these actions may be direct or indirect—direct if the curative change is produced in the part upon which the action of the remedy is exerted; indirect if the change is brought about in an organ not acted on by the remedy.

Elective remedies must enter the circulation or other of the internal fluids, in order that their action may be manifested. It is not necessary for certain remedies to enter the blood directly to produce their action upon certain parts, but may enter the chyle or intestinal fluids, and in this latter way the most speedy effect is produced upon certain parts. For instance, when chloroform or a prepation of aconite or opium is rubbed on the skin at any part, as soon as it has had time to permeate the cuticle, it paralyzes the sentient nerves. These are bathed in the intestinal fluids of the part.

Thus we see that it is not necessary for them to enter the circulation at all to produce their effect upon these nerves, though when applied in large amounts it enters the circulation and produces its effects upon distant parts. Thus belladonna or atropine, which, when applied to the surface of the eye or neighborhood of the orbit, influences the nerves of the pupil in such a manner as to cause the latter to dilate, or the principle of calabar bean, which causes it to contract, must in all probability find a more direct road to these nerves than that which is offered by the vascular system.

Dr. W. F. Westmoreland, from experiments made upon chickens and other fowls by the bite of rattlesnakes, seems to think that the poison may be transmitted to the nerve centers by means of the nerves themselves. In the experiments made by him, there was no perceptible time elapsed between the blow of the serpent and the death of the animal. Death was instantaneous with the blow or bite.

There are, however, four substantial reasons for believing that it is necessary for the manifestation of the action of an elective remedy that it shall enter the blood or other of the internal fluids of the body.

1. When introduced into the circulation at any other point, they act in the same way as when taken into the stomach.

2. The continuity of a nerve is not necessary for them to exert their action, but the vascular connection is necessary.

3. The circulation of the blood is sufficiently quick to account for the effect of those poisons which act most rapidly by influencing the nerve centres.

4. The great majority of elective remedies have been found in the blood and the secretions formed out of it.

While, perhaps, no one of the above propositions is positive proof of our position, yet, taken altogether, it seems to establish, beyond a doubt, the truthfulness of our statement: that while some of the neurotics may act through chemical process, it is absolutely necessary that elective remedies enter either the circulation or other internal fluids of the body, in order that their physiological action may be had. But we repeat, that it is not necessary for all neurotics to enter the blood and be conveyed to the nervous centres before their physiological action, or at least the effect of their action, can be exerted. If this were absolutely necessary, why does not a solution of the sulphate of atropia, when dropped upon the ball of one eye, cause the pupil of the opposite eye to become dilated as quickly and completely as that of the eye in which it was placed?

Some elective remedies do, however, exert a slight action upon the membrane through which it is absorbed, as a mucous membrane, and it may also act upon certain glands as it makes its exit from the circulation. Yet, the prime action—that to which most importance should be attached—is had by these elective or constitutional remedies while in the blood or in the tissues upon which their physiological action is exerted.

There are many circumstances which modify the action of remedies, some to a greater, some to a less extent.

It is highly important for the medical practitioner to understand these conditions or circumstances; for serious results have come of the administration of a single dose of medicine to a patient in whom there was a peculiar susceptibility to the action of the remedy, when in the great majority of cases no such bad results would have followed.

It is not necessary for us to enumerate all these circumstances, but will be content to mention a few of the most important.

1. Climate modifies, to a certain extent, the physiological action of remedies. Mercurials given for the purpose of exciting the liver, are required in larger quantity in the South than in the North. Again, persons reared in a Southern climate are said to be more susceptible to the action of opiates, than those residing in a Northern climate. This is also true of calorifics—heat producing agents—requiring a much smaller quantity to keep up the requred temperature of the body in the South than in the North.

2. Sex. While in the majority of instances but little or no difference in the dose administered to the male and female is to be observed, yet the greater susceptibility of the female to mental influences and nervous impressions, should cause us to be more guarded in the use of neurotic medicines than in the stronger sex. The changes produced in the system by the functions of the uterus, modify the action of remedies, and, not unfrequently, cause us to adopt a mode of treatment altogether different from that which we would adopt if such physiological conditions did not exist. No one would think of administering ergot or drastic cathartics to a pregnant female in the treatment of diseases, which, under other circumstances, would indicate the use of such remedies.

3. Habit. This should not be lost sight of in the administration of remedies; for it often modifies the action of remedies. In the majority of cases, it renders the system less susceptible to the impression of certain remedies, requiring for their ordinary or natural action a larger amount than the usual quantity administered to produce the desired result. The "opium eater" and the "whisky drinker" would be but slightly effected, if at all, by the usual dose or quantity of these stimulants. Not only does habit in this respect modify the action of remedies, but it is true of all his habits, the manner of living, the character of exercise, etc. The adage that "habit is second nature," is as true in a medical point of view as in any other respect.

4. Idiosyncrasy. This is a constitutional confirmation, which prevents the remedy from exerting its physiological action upon the system. It is generally an easy matter to acquaint oneself with the idiosyncrasy (if it exists) of each patient, and at no time should the importance of the truth that this peculiarity in the constitution modifies materially the action of remedies, be overlooked. It not unfrequently occurs that remedies administered to patients possessing this peculiar confirmation, produce a result just opposite to that which is known to be its usual effect.

5. Incompatibility of remedies. This often interferes with their physiological action. It is known that certain remedies, perfectly innocent within themselves, when combined become virulent poisons; while the potency of some of our most active remedies, by combination with other remedies, is destroyed—becoming perfectly inert and worthless in the treatment of disease. There are, as stated elsewhese, however, chemical, not physiological incompatibles. In the living body there are many conditions which interfere with the changes which would take place in the laboratory of the chemist.

6. Temperament. There are four temperaments—the lymphatic, nervous, sanguine and bilious, each of which should be definitely taken into consideration in order to calculate with certainty the degree of physiological action to be effected by certain remedies and the therapeutic changes produced by such action. In the sanguine temperament, depletion and antiphlogistic treatment can be carried to almost any extent desired; while such a course with the lymphatic, if persisted in, would soon carry the patient to his grave. Where the nervous influences predominate, exciting neurotics should be administered with caution.

7. Age. This possesses a greater modifying influence over the action of remedies than any other circumstance. It is not sufficient, in many instances, to administer a quantity proportionate to the age compared with that for an adult, for this modifying influence exists independently of the difference of age. We know that a dose of opium, proportionate to the dose for an adult, if administered to a child, will produce even alarming effects. It is known, also, that children are much less subject to the action of mercurials, in proportion to their age, than adults. A quantity which would perhaps be sufficient to produce ptyalism in the adult, will produce no such results upon the child. Why this difference should exist, or upon what peculiarity it depends, is but little understood. Dr. J. G. Westmoreland, in his "Acology and Therapeutics," says upon this point: "Whether this depends upon a want of susceptibility in the parts to be acted on, a deficiency of solvent material in the stomach, or a want of activity in the absorbents, is not very clearly understood."

This difference in the action of remedies is not confined to childhood. Remedies which, in youth and prime of life would produce but slight impressions upon the system, would often produce alarming effects upon the aged. This susceptibility to the action of remedies in old age should be known, and great caution exercised in the administration of remedies to this class of patients.

To Dr. J. G. Westmoreland the medical profession and the public in general are indebted for instituting a rational and scientific system for the correct studying of the physiological action of remedies. He stated to the present medical class that he was proud to say that most new works on this subject are adopting a classification founded upon this action.

